# A GHKNN model based on the physicochemical property extraction method to identify SNARE proteins

**DOI:** 10.3389/fgene.2022.935717

**Published:** 2022-11-23

**Authors:** Xingyue Gu, Yijie Ding, Pengfeng Xiao, Tao He

**Affiliations:** ^1^ State Key Laboratory of Bioelectronics, School of Biological Science and Medical Engineering, Southeast University, Nanjing, China; ^2^ Yangtze Delta Region Institute (Quzhou), University of Electronic Science and Technology of China, Quzhou, Zhejiang, China; ^3^ Institute of Fundamental and Frontier Sciences, University of Electronic Science and Technology of China, Chengdu, China; ^4^ Beidahuang Industry Group General Hospital, Harbin, China

**Keywords:** SNARE proteins, GHKNN, SMOTE, identify protein sequences, physicochemical property extraction method

## Abstract

There is a great deal of importance to SNARE proteins, and their absence from function can lead to a variety of diseases. The SNARE protein is known as a membrane fusion protein, and it is crucial for mediating vesicle fusion. The identification of SNARE proteins must therefore be conducted with an accurate method. Through extensive experiments, we have developed a model based on graph-regularized k-local hyperplane distance nearest neighbor model (GHKNN) binary classification. In this, the model uses the physicochemical property extraction method to extract protein sequence features and the SMOTE method to upsample protein sequence features. The combination achieves the most accurate performance for identifying all protein sequences. Finally, we compare the model based on GHKNN binary classification with other classifiers and measure them using four different metrics: SN, SP, ACC, and MCC. In experiments, the model performs significantly better than other classifiers.

## 1 Introduction

SNAREs mediate most of the intracellular membrane fusion events, and mammalian cells have over 30 members of the SNARE family, each found in a different subcellular compartment (Jahn and Scheller, 2006; van Dijk et al., 2008). SNAREs may encode aspects of membrane transport specificity, but the mechanisms by which they achieve specificity remain controversial (Ferro-Novick and Jahn, 1994; Rothman, 1994). Studies have shown that SNAREs are targets of CNT proteases, thus establishing the importance of SNARE proteins for synaptic neurotransmission (Schiavo et al., 1992; Blasi et al., 1993; Schiavo et al., 1993; Yamasaki et al., 1994a; Yamasaki et al., 1994b; Schiavo et al., 1995). Therefore, the accurate identification of SNARE proteins is particularly necessary and important. Up to now, experimenters have used a number of methods to identify SNARE proteins from a biological perspective. Traditional biological experiments have the disadvantage of long lead times and high costs. Machine learning and data mining have ushered in a new era of protein prediction (Xiong et al., 2012; Cao et al., 2014; Wei et al., 2014; Chen et al., 2016; Ding et al., 2016; Wei et al., 2016; Zou et al., 2016; Zeng et al., 2017a; Zhang et al., 2017; Xiong et al., 2018a; Bu et al., 2018; Liao et al., 2018; Wei et al., 2018; Cao et al., 2019; Chao et al., 2019; Chen et al., 2019; Le and Nguyen, 2019; Liu et al., 2019; Małysiak-Mrozek et al., 2019; Meng et al., 2019; Ghulam et al., 2020; Guo et al., 2021; Qian et al., 2022; Tiwari et al., 2022).

In this paper, a classification model based on the GKHNN algorithm is adopted to accurately identify SNARE proteins. Three datasets are used: a cross-training dataset, an independent-validation dataset, and the all-dataset containing all samples. To obtain 188-dimensional sample attribute features, the physicochemical property extraction method was used in this study to extract the sample features from three datasets. The sample sets of the three datasets were very unbalanced. In order to minimize the interference of the unbalanced datasets on the binary classification accuracy, this experiment uses the SMOTE upsampling method to make the positive and negative samples balanced. The GKHNN classifier model performs binary classification on each of the three datasets while comparing the classification results with the 2DCNN algorithm. Meanwhile, the binary classification experimental results are compared with other four classifiers on the complete dataset to verify the high accuracy of this model in classifying SNARE proteins; different feature extraction methods are used to compare the experimental results obtained using the 188D feature extraction method selected in this experimental model to verify the effectiveness of this experimental model feature extraction method; two other protein datasets in this field are selected for classification, and the generalizability of this experimental model was verified by comparing with previous experiments. This study uses four measures, namely, accuracy (ACC), sensitivity (SN), specificity (SP), and the Mathews correlation coefficient (MCC), to measure the degree of accuracy of the algorithmic model classification.

The structure and content of this paper are as follows: Section 1 describes the importance of identifying SNARE proteins as well as the structure and distribution of this paper. Section 2 describes the construction of the experimental dataset, preprocessing, and number of samples, as well as the specific experimental procedure of this experiment. Also, the physical and chemical property extraction method, the SMOTE dataset balancing method, and the classifier algorithm GKHNN are described in detail. Section 3 describes the comparison of experimental results when the specific parameters of this experimental model are taken at different values, the comparison of experimental results between this experimental model and other classifiers, the comparison of experimental results using the 188D feature extraction method used in this experiment and the four other common feature extraction methods, and the comparison of experimental results when this experimental model is applied to other datasets. The discussion of the current work is given in Section 4.

## 2 Materials and methods

### 2.1 Data retrieval and pretreatment

#### 2.1.1 Dataset

Datasets were collected from the UniProt database (Consortium, 2015), which is one of the most comprehensive database resources for protein sequences. First, all proteins annotated with the keyword “snare” were collected from the UniProt database. It is worth noting that the proteins collected were all reviewed (extracted from the literature and assessed by the administrator for calculation and analysis). Subsequently, more than 30% of the redundant sequences were removed by the BLAST database (Altschul et al., 1997). After this process, only 245 SNARE proteins remained, and the number of proteins was not sufficient to build an accurate deep learning model. Therefore, we used a truncation level of 100% in the cross-training dataset to build a significant model. In the independent dataset, we still used a 30% similarity level to assess the classification performance of the model. This is critical for testing the model (Le and Nguyen, 2019).

In order to build a classification model with high classification accuracy, the dataset has a crucial role to play. The negative dataset collected should be similar to the positive dataset in terms of structure and function of the proteins. After considering the structure and function of the positive protein sequence, SNARE, the vesicle transporter protein, which is a general protein that includes the SNARE protein, was chosen as the positive dataset for this experiment. Thus, the problem was transformed into a binary classification problem for SNARE proteins and vesicular transporter proteins (vesicular transport proteins are referred to as non-SNARE proteins). We removed redundant datasets and those with greater than 30% similarity between the two datasets. Finally, we divided the data into cross-training, independent-validation, and the all-dataset. The details of the three datasets used in this study are shown in Table 1.

**TABLE 1:** Number of raw SNARE and non-SNARE proteins in the cross-training dataset, independent-validation dataset, and all-dataset.

Dataset	Cross-training	Independent-validation	All-dataset
SNARE	644	38	682
Non-SNARE	2,234	349	2,583

#### 2.1.2 Experimental procedure

The specific process for this experiment is as follows:

(1) After obtaining the data shown in Table 1, the cross-training dataset, the independent-validation dataset, and the negative and positive samples in the all-dataset were extracted using the physicochemical property extraction method to obtain 188-dimensional sample attribute features, respectively. In the cross-training dataset, there are 644*188-dimensional SNARE protein attributes and 2,234*188-dimensional non-SNARE protein attributes. In the independent-validation dataset, there are 38*188-dimensional SNARE proteins and 349*188-dimensional non-SNARE proteins. In the all-dataset, there are 682*188 dimensional SNARE proteins and 2,583*188 dimensional non-SNARE proteins.
(2) Due to the high imbalance in the number of positive and negative samples in the dataset, the cross-training dataset, the independent-validation dataset, and the all-dataset were upsampled separately using SMOTE. The number of positive and negative samples for the cross-training dataset was 2,200 and 2,234, respectively; the number of positive and negative samples for the independent-validation dataset was 350 and 349; and the number of negative and positive samples for the all-dataset was 2,550 and 2,583, respectively. This resulted in a balance of negative and positive samples. The specific numbers of negative and positive samples in the three datasets are shown in Table 2.
(3) Positive and negative samples from the independent-validation dataset, the cross-training dataset, and the all-dataset were classified using the GKHNN classifier and measured using four metrics based on specificity (spec), sensitivity (recall), Matthews correlation coefficient (mcc), and accuracy (acc). Specific results on the accuracy of the classification model are given in detail in Section 3.

**TABLE 2:** Number of SNARE and non-SNARE proteins in the cross-training dataset, the independent-validation dataset, and the all-dataset after SMOTE equilibration.

Dataset	Cross-training	Independent-validation	All-dataset
SNARE	2,200	350	2,550
Non-SNARE	2,234	349	2,583

**FIGURE 1:**
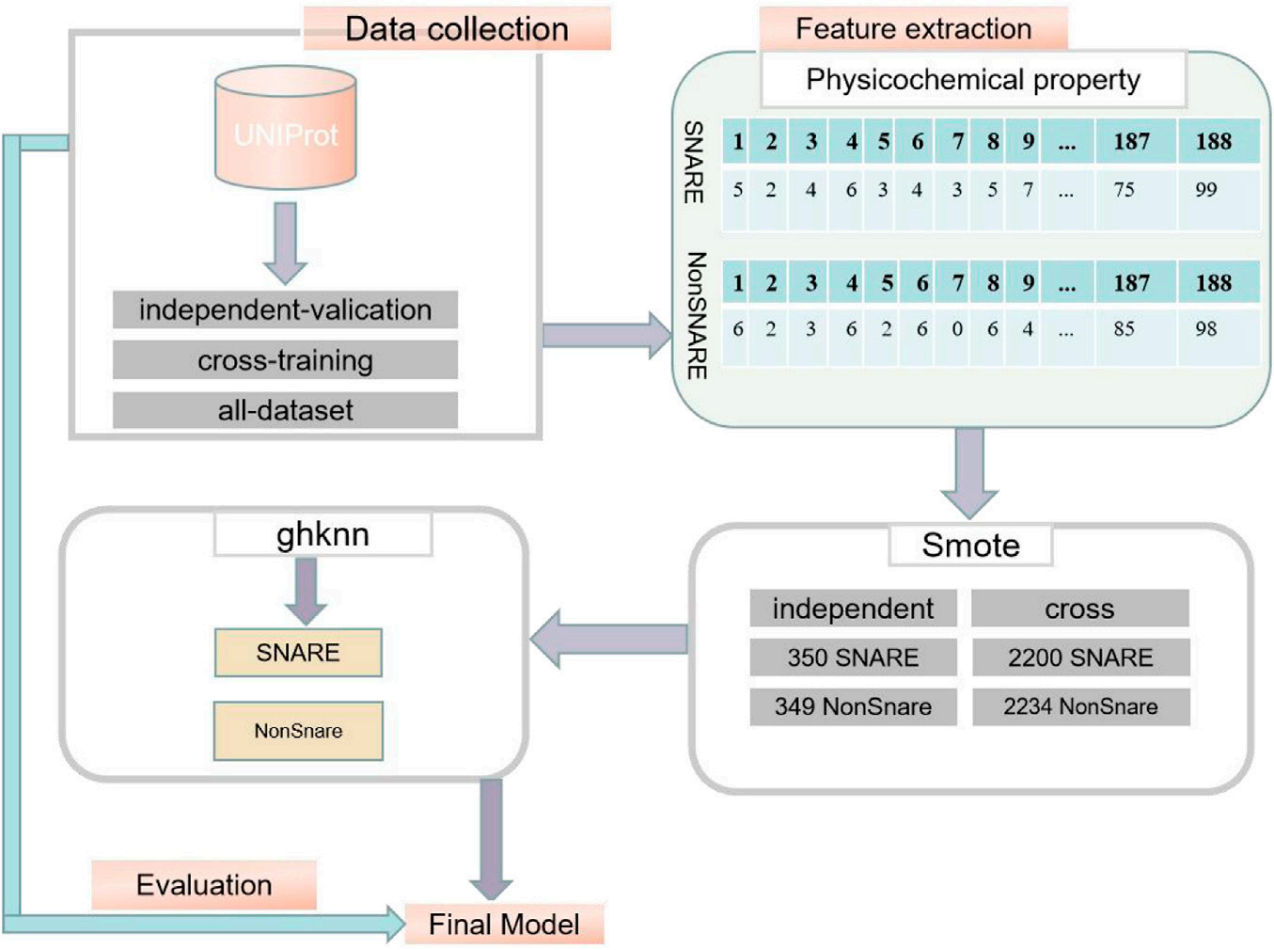
Experimental procedure diagram.

The exact procedure of the experiment is shown in Figure 1.

### 2.2 Physicochemical property extraction method

To extract sample features, we used the physicochemical property extraction method. The composition and position of protein molecules as well as its physicochemical characteristics have been used by previous researchers to extract protein features (Dubchak et al., 1995; Shen et al., 2017; Wang et al., 2017; Yu et al., 2017; Xiong et al., 2018b; Qiao et al., 2018; Zhang et al., 2018; Shen et al., 2019; Zou et al., 2019; Liu et al., 2020). Cai et al. (2003) used a physicochemical property feature extraction method to extract protein features, where the composition, distribution, and physical properties of amino acids were included. In one category, there are 20 amino acid features denoted as F1...F20, calculated using the following Eq. 1:

$$F_{i}=\frac{n_{i}}{L}\left(i=1,...,20\right).$$ (1)

Here, the 20 features are represented by F, L denotes the length of the protein sequence, and $n_{i}$ represents the frequency of each amino acid.

The other category is the physicochemical properties represented by 168 features, which are extracted from eight physicochemical properties of the protein, including polarity, secondary structure, polarizability, normalized van der Waals volume, and hydrophobicity. Each property has 21 features, for e.g., the 21 features of polarity are represented by F21–F41; hydrophobicity features are represented by F42–F52, calculated using the following Eq. 2:

$$\left(F_{21},F_{22},F_{23}\right)=\left(\frac{CA_{1}}{L},\frac{CA_{2}}{L},\frac{CA_{3}}{L}\right).$$ (2)

According to the surface tension, the 20 amino acids are divided into three groups (Cai et al., 2003). Therefore, ${\text{CA}}_{1}$ , ${\text{CA}}_{2}$ , and ${\text{CA}}_{3}$ denote the content of the three groups, respectively.

$$\left(F_{24},...F_{28};F_{29},...F_{33};F_{34},...F_{38}\right)=\left(\frac{DA_{11}}{L},...\frac{DA_{15}}{L};\frac{DA_{21}}{L},...\frac{DA_{25}}{L};\frac{DA_{31}}{L},...\frac{DA_{35}}{L}\right),$$ (3)

where ${\text{DA}}_{\text{ij}}$ (the range of values for i is from 1 to 3 and the range of values for j is from 1 to 5) indicates the position of the first 25%, 50%, and 75% of the chain length of the AAs of the GQDNAHR group among the three groups (Zou et al., 2013).

$$\left(F_{39},F_{40},F_{41}\right)=\left(\frac{FA_{1}}{L-1},\frac{FA_{2}}{L-1},\frac{FA_{3}}{L-1}\right).$$ (4)

Here, the numerator ${\text{FA}}_{i}$ (i = 1,2,3) indicates the number of bivalent seeds in different groups, where bivalent seeds contain two AAs. The denominator L-1 denotes the number of bivalent seeds.

This results in 20 features of amino acid composition and 21 features represented by each of the eight physicochemical properties of the protein, for a total of 168 features. This gives a total of 188 protein features (Gao and Li, 2020).

### 2.3 Sampling method

#### 2.3.1 SMOTE upsampling technique

The negative and positive samples in the cross-training dataset, the independent-validation datasets and all-datasets are: 2,234, 349, and 644; 38,682, and 2,583, respectively. The positive and negative samples are quite unbalanced. To calculate the k nearest neighbors of an instance x in each of the few classes, the Euclidean distance between the instance x and the other instances in that class is calculated using SMOTE algorithm. The sampling rate N is determined by the imbalance rate. Several instances are chosen from the k nearest neighbors of x (Gao and Li, 2020). Finally, the following Eq. 5 is used to build an instance $x_{new}$ based on x and $x_{n}$ :

$$x_{new}=x+rand\left(0,1\right)*|x-x_{n}|.$$ (5)

### 2.4 Graph-regularized k-local hyperplane distance nearest neighbor model

Although HKNN algorithm incorporates a local hyperplane classification algorithm for better classification prediction performance, it is still in the original input space in terms of feature measurement. Its objective function is the distance from the sample x to the *c*th hyperplane:

$${(\text{LH}}_{k}^{c}{\left(x)\right)}^{2}={\left\|x-\overset{-}{N^{c}}-\overset{k}{\underset{i=1}{\sum }}\alpha_{i}^{c}V_{i}^{c}\right\|}^{2}+\lambda \overset{k}{\underset{i=1}{\sum }}{(\alpha_{i}^{c})}^{2}),$$ (6)

$$\alpha^{c}(\lambda I+V^{c}(V^{c}{)}^{T})=(x-\overset{-}{N^{c}}){\left(V^{c}\right)}^{T},$$ (7)

where H in Eq. 6 denotes the hyperplane; k is the nearest neighbor, which refers to the nearest sample threshold of the test sample x; V denotes the matrix consisting of k nearest neighbor sample vectors of the test sample x; and N is the center of mass of the k nearest samples to the sample point x.

The GHKNN introduces kernel learning techniques and graph regularization terms to improve the generalization ability of the model and the association between samples. If the dimensionality of the original feature space is low, the model is not able to find a reasonable classification hyperplane. Therefore, by mapping the features in the original space to a suitable high-dimensional feature space and solving (6) using the kernel technique, the model is not able to find a reasonable classification hyperplane as the dimensionality of the original feature space is low, so it needs to be spatially projected to a high-dimensional space to find a reasonable classification hyperplane. Let x be mapped to f (let φ:x→F ) and $\bar{x}=x-\bar{N^{c}}$ , which is the de-priming of the original data that serve to reduce the influence of noisy samples on the model. (6) can be reformulated as:

$$\arg\min_{\alpha^{c}}(LH_{k}^{c}{\left(x)\right)}^{2}=\lambda \overset{k}{\underset{i=1}{\sum }}{\left(\alpha_{i}^{c}\right)}^{2}+\mu \overset{k}{\underset{p=1}{\sum }}\overset{k}{\underset{q=1}{\sum }}\omega_{p,q}^{c}{\left(\alpha_{p}^{c}-\alpha_{q}^{c}\right)}^{2}+{\left\|\varphi \left(\overset{-}{x}\right)-\overset{k}{\underset{i=1}{\sum }}\varphi \left(V_{i}^{c}\right)\partial_{i}^{c}\right\|}^{2},$$ (8)

where φ(x) is the invisible mapping function, ω refers to the weight between samples, and μ is the regularization factor.

In order to solve the parameters of the model, let the derivative of the left-hand side of the equation is 0 and $\frac{\partial ((LH_{k}^{c}{\left(x)\right)}^{2})}{\partial \alpha^{c}}=0,\alpha^{c}$ , we obtain:

$$\partial \left[\lambda \alpha^{c}(\alpha^{c}{)}^{T}+(\varphi (\overset{-}{x})-\varphi (V^{c})\alpha^{c})(\varphi (\bar{x})-\varphi (V^{c})\alpha^{c}{)}^{T}\right]+\mu trace(L\alpha^{c}\left(\alpha^{c}{)}^{T}\right)]/\varphi \alpha^{c}=0,$$ (9a)

$$-\varphi (V^{c}{)}^{T}\varphi (\bar{x})+\varphi (V^{c}{)}^{T}\varphi (V^{c})\alpha^{c}+\lambda \alpha^{c}+\mu L\alpha^{c}=0,$$ (9b)

$$\left(\varphi (V^{c}{)}^{T}\varphi (V^{c})+\lambda I+\mu L)\alpha^{c}=\varphi (V^{c}{)}^{T}\varphi (\bar{x}\right),$$ (9c)

$$\alpha^{c}=\frac{\varphi (V^{c}{)}^{T}\varphi (\bar{x})}{\left(\mu L+\lambda I+\varphi {\left(V^{c}\right)}^{T}\varphi \left(V^{c}\right)\right)},$$ (9d)

$$\alpha^{c}=\frac{K\left(V^{c},\bar{x}\right)}{\left(\lambda I+K\left(V^{c},V^{c}\right)+\mu L\right)},$$

where $K\left(V_{c},V_{c}\right)$ is a positive semi-definite lattice matrix for RBF calculations of k*k dimensions and $K\left(V_{c},\bar{x}\right)$ is a vector of k*1 dimensions. The inner product form represents the kernel matrix, and the RBF kernel matrix is calculated as follows:

$$K\left(x_{i},x_{j}\right)=\exp\left(-\gamma {\left\|x_{i}-x_{j}\right\|}^{2}\right),$$ (10)

where γ indicates the bandwidth (the variance of the Gaussian distribution), $x_{i}$ and $x_{j}$ are the eigenvectors of sample i and sample j, respectively, and the RBF values of these two samples are obtained by an exponential function with e as the base.

The following equation calculates the distance between the *c*th hyperplane and the test sample x. Here, $p^{c}$ denotes the *c*th hyperplane and dist_c is the distance from sample x to the hyperplane of category c. Since a high-dimensional projection is involved, the kernel function trick is used to convert the inner product operation of the vector into the RBF value operation of the sample.

$$\begin{array}{l} {\text{dist}}_{c}=\text{dist}\left(x,p^{c}\right)\\ ={\left\|\varphi \left(x-\overset{-}{N^{c}}\right)-\varphi \left(V^{c}\right)\alpha^{c}\right\|}^{2} \end{array}$$

$$=(\varphi (\overset{-}{x})-\varphi (V^{c})\alpha^{c})(\varphi (\overset{-}{x})-\varphi (V^{c})\alpha^{c}{)}^{T}$$ (11)

$$=K(\overset{-}{x},\overset{-}{x})+\alpha^{c}(\alpha^{c}{)}^{T}K(V^{c},V^{c})-2K\left(V^{c},x\right){\left(\alpha^{x}\right)}^{T}.$$

Finally, when assigning the test sample x to class c, the following results are obtained (Sun et al., 2021; Ding et al., 2022):

$$\text{class}c=\min\text{dist}\left(x,p^{c}\right),c=1,2,...C.$$ (12)

## 3 Results

### 3.1 Model assessment

This experiment used several commonly used evaluation metrics to measure the accuracy of model classification, including sensitivity (SN) (Chou, 2001; Chou, 2011; Lai et al., 2017; Liu et al., 2017; Ding et al., 2022), specificity (SP), accuracy (ACC), and Matthews correlation coefficient (MCC) (Matthews, 1975; Yu et al., 2015; Zeng et al., 2017b; Wei et al., 2017; Jia et al., 2018; Zhang et al., 2019a; Zhang et al., 2019b; Shan et al., 2019; Zeng et al., 2019; Hong et al., 2020). The four evaluation indicators are given in the following four formulas:

$$SN=\frac{TP}{FN+TP},$$ (13)

$$SP=\frac{TN}{FP+TN},$$ (14)

$$MCC=\frac{TP*TN-FP*FN}{\sqrt{\left(FN+TN\right)\left(EN+TP\right)\left(FP+TN\right)\left(FP+TP\right)}},$$ (15)

$$\text{ACC}=\frac{TN+TP}{FN+FP+TN+TP}.$$ (16)

A false-positive, a true-negative, a false-negative, and a true-positive are, respectively, referred to as FP, TN, FN, and TP (Hou et al., 2020).

### 3.2 Parameter adjustment

**FIGURE 2:**
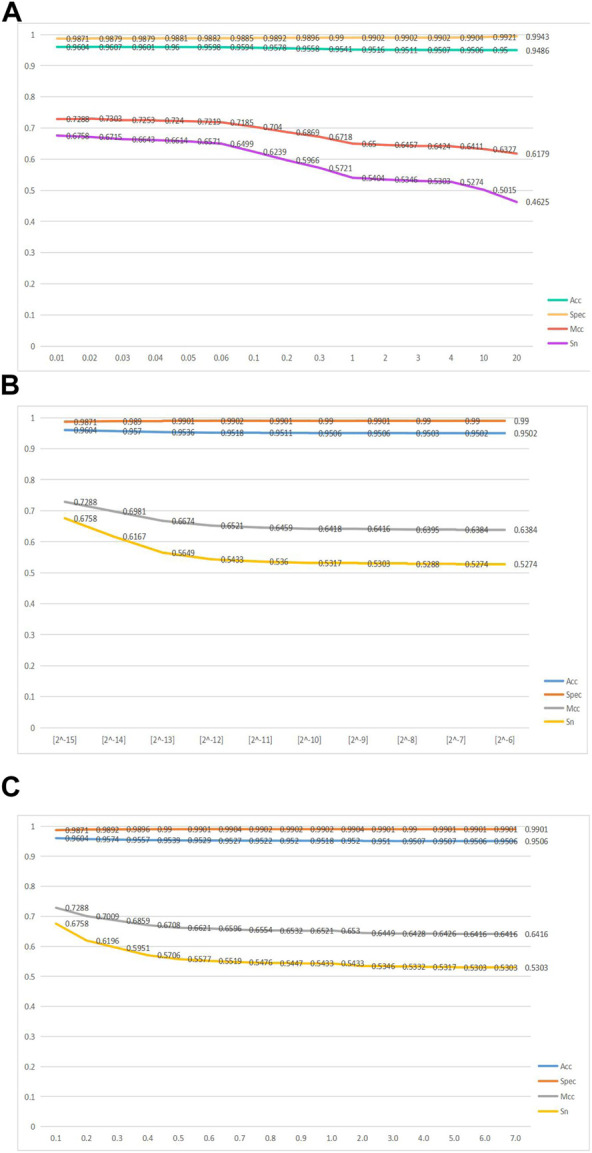
Line graphs of the four metrics, namely, acc, spec, mcc, and sn, as the variable parameters λ, γ , and μ change. **(A)** Line graph of Acc, Spec, Mcc, and Sn of GHKNN with the change in parameter λ . **(B)** Line graph of Acc, Spec, Mcc, and Sn of GHKNN with the change in parameter γ . **(C)** Line graph of Acc, Spec, Mcc, and Sn of GHKNN with the change in parameter μ .

The GKHNN classifier has three variable parameters: λ, γ, and μ . Figure 2 represents line graphs of the four metrics, acc, spec, mcc, and sn, as the variable parameters λ, γ , and μ change.

Figure 2A shows a line graph of the GKHNN classifier bifurcating the positive and negative sample sets as the parameter λ increases from an initial value of 0.01 to 20, with the change in four metrics, acc, spec, mcc, and sn, when μ is 0.1 and γ is [2^-6]. It can be seen from the figure that, with the continuous increase in λ , the four indicators, acc, spec, mcc, and sn, all declined. Among them, mcc and sn dropped significantly, and the spec line showed a gentle upward trend. Mcc decreased from 0.7288 to 0.6179; sn decreased from 0.6758 to 0.4625; the acc and spec indicators, on the other hand, were less affected by the increase, with the acc values falling more gently but still decreasing; and the spec line showed a slightly rising trend. Therefore, from Figure 2A, it can be seen from the four indicators that when λ is at a minimum value of 0.01, the model has the best classification ability on positive and negative sample sets (Zou et al., 2017; Zhao, 2020; Zhu et al., 2021; Zou, 2021).

Figure 2B shows a line graph of the four metrics, acc, spec, mcc, and sn, continuously changing as the parameter γ rises from the initial value [2^-15] to [2^-6] and the GKHNN classifier bifurcates the set of positive and negative samples, when μ is 0.1 and λ is 0.01. The graph shows that the three indicators, acc, mcc, and sn, all decrease in the course of the rise in the index, while spec increases very slowly; the mcc and sn folds decrease with a clear slope, with sn decreasing from 0.6758 to 0.5274 and mcc decreasing from 0.72880 to 0.6384, while acc is less affected by the change, with a more gentle slope of the folds but still shows a decreasing trend. Figure 2B, combining the four metrics, shows that the model performs best in terms of classification ability on the positive and negative sample sets when γ takes the maximum value [2^-15].

Figure 2C shows a line graph of how the four metrics, acc, spec, mcc, and sn, change as μ rises from an initial value of 0.1 to 7 for the GKHNN classifier for positive and negative sample set binary classification, when λ is 0.01 and γ is [2^-6]. The graph shows that as μ continued to increase, all three indicators, acc, mcc, and sn, declined, and the spec curve gradually increased. The slope of the decreasing curve of mcc and sn is obvious, with mcc decreasing from 0.7288 to 0.6416 and sn decreasing from 0.6758 to 0.5305, while two other indicators, acc and spec, are less affected by the increasing μ value. Figure 2C, combining the four metrics, shows that the model performs best in terms of classification ability on the positive and negative sample sets when μ takes the minimum value of 0.1.

Ultimately, based on the aforementioned line graphs, it was concluded that GKHNN classification performed best when the values of 0.01, [2^-15], and 0.1 were set for λ, γ , and μ , respectively. The specific values taken are shown in Table 3.

**TABLE 3:** Values of parameters α,μ,γ of the GHKNN classifier.

Parameter	λ	γ	β
Parameter values	0.01	[2^-15]	0.1

### 3.3 Comparison with other methods

Table 4 shows the comparison of the classification results of GKHNN with 2DCNN on the all-dataset, the independent-validation dataset (Iv dataset), and the cross-training dataset (Ct dataset). As shown in the table, on the Ct dataset, the classification result values of this experimental model are higher than those of 2DCNN; on the Iv dataset, only the experimental result value of Sn is slightly lower than that of 2DCNN, and the other three metrics are higher than those of 2DCNN. Therefore, it seems that the present experimental model outperforms 2DCNN in terms of binary classification on these datasets in a comprehensive manner.

**TABLE 4:** Values of Sn, Acc, Spec, and Mcc of the GHKNN and 2DCNN classifiers on the Iv dataset and Ct dataset.

	Ct dataset				Iv dataset			
	Spec	Acc	Mcc	Sn	Spec	Acc	Mcc	Sn
GHKNN	0.969	0.934	0.806	0.814	0.946	0.900	0.470	0.588
2DCNN	0.935	0.897	0.7	0.766	0.903	0.897	0.460	0.658

**FIGURE 3:**
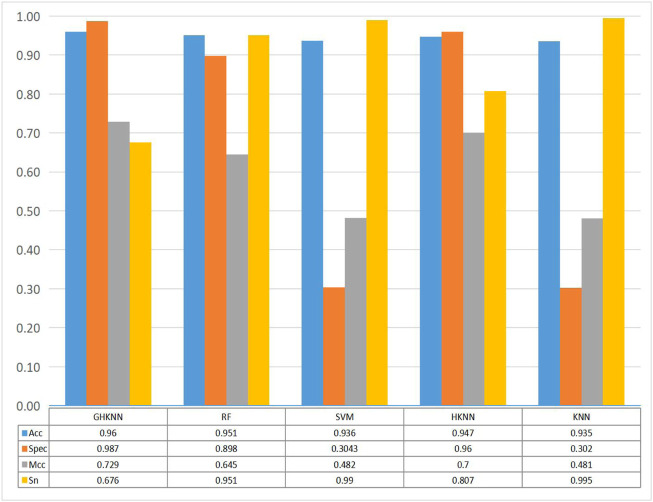
Histograms of the four metrics, namely, sn, acc, spec and mcc, obtained from the classification of the dataset by the five classifiers: GHKNN, RF, SVM, HKNN, and KNN.

Figure 3 shows the comparison of the classification effect of the GHKNN classifier with four other classifiers: random forest (RF), support vector machine (SVM), k-local hyperplane distance nearest neighbor (HKNN), and k nearest neighbor (KNN), on the three datasets. As can be seen from the graph, the Acc, Spec, and Mcc values of GHKNN are higher than those of the other four classifiers, and only the Sn value is slightly lower.

Collectively, it seems that the classification effect of the GHKNN classifier exceeds that of the other classifiers, and thus, this experimental model has the best classification effect on the three datasets.

### 3.4 Comparison with other feature extraction methods

Table 5 shows the experimental comparison results of the 188D feature extraction method used in this experiment with four other common feature extraction methods with representative values: AAC, CTDC, GAAC, and CKSAAGP. From the table, it can be seen that the values of Acc, spec, mcc, and sn of the 188D feature extraction method used in this experiment are much higher than those of the other four extraction methods

**TABLE 5:** Comparison of Sn, Acc, Spec, and Mcc values obtained by using GHKNN-based classification on this experimental dataset using five extraction feature methods: 188D, AAC, CTDC, GAAC, and CKSAAGP.

	Acc	Spec	Mcc	Sn
188D	0.960	0.987	0.729	0.676
AAC	0.936	0.990	0.495	0.350
CTDC	0.925	0.989	0.388	0.252
GAAC	0.903	0.976	0.172	0.134
CKSAAGP	0.939	0.987	0.540	0.420

### 3.5 Comparison with previous classification results on other datasets

Table 6 shows the results of this experimental model on the G protein-coupled receptor (GPCR) dataset compared with those of Liao et al. (2016) and on the vesicular transport protein (vesi) dataset compared with those of Le and Nguyen (2019). As can be seen from the table, on the GPCR dataset, the values of the present experimental model is lower than the predicted value of Liao only on spec, which is about 0.04, and outperforms Liao on all three measures of acc, mcc, and sens; on the vesi dataset, the values of the present experimental model is lower than those of Nguyen on the index of sens, and the other three measures are higher than those of Nguyen. Collectively, it seems that the present experimental model outperforms the previous experimental results in classification on both GPCR and vesi datasets.

**TABLE 6:** Classification results using this experimental model on the GPCR and vesi datasets and the comparison with the previous experimental results.

	GPCR				Vesi			
	Spec	Acc	Mcc	Sn	Spec	Acc	Mcc	Sn
GHKNN	0.937	0.934	0.865	0.930	0.952	0.879	0.623	0.618
Others	0.972	0.833	0.692	0.694	0.829	0.823	0.520	0.792

## 4 Discussion

This study shows that the GKHNN-based binary classifier has good classification results for proteins. Compared to several other classifiers, the four metrics, spec, recall, mcc, and acc, reached high values on the three datasets of this study, which have some application value. In the study of the binary classification problem of protein sequences, both in the field of machine learning and deep learning, the classification accuracy of protein sequences is highly variable due to the individual performance of each protein sequence, resulting in significant differences in the classification results using different classifiers. As a result, we are committed to finding more general classification models with wider applicability in the future.

## Data Availability

Publicly available datasets were analyzed in this study. These data can be found at: https://github.com/ gugu131300/GHKNN_code.
