# Supplementary figures and images for: A GHKNN model based on the physicochemical property extraction method to identify SNARE proteins
Source: Front Genet. 2022 Nov 23;13:935717. doi: 10.3389/fgene.2022.935717 (PMC9727185; doi:10.3389/fgene.2022.935717)

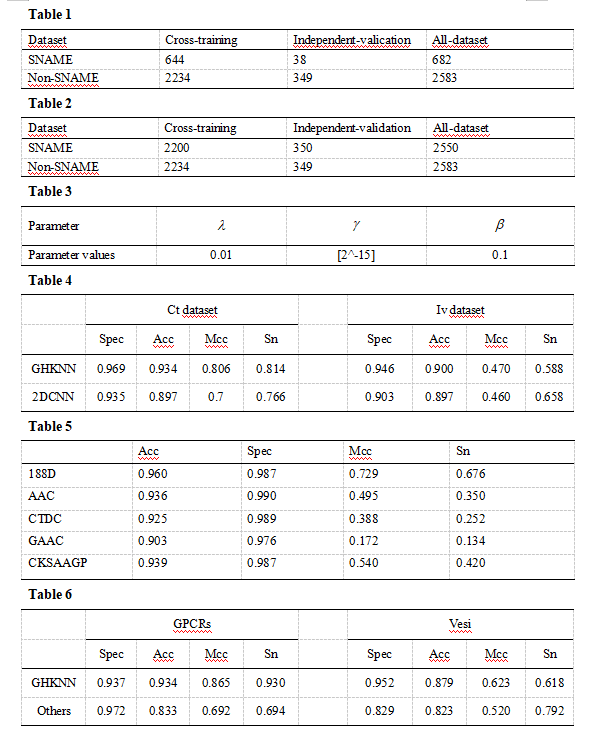

Supplement: Supplementary file 1 [file Image1.PNG]
